# Temporomandibular Disorders and Fibromyalgia Prevalence: A Systematic Review and Meta-Analysis

**DOI:** 10.11607/ofph.3260

**Published:** 2023-11-17

**Authors:** Pankaew Yakkaphan, Jared G. Smith, Pav Chana, Huann Lan Tan, Priya Thimma Ravindranath, Giorgio Lambru, Tara Renton

**Affiliations:** Faculty of Dentistry, Oral & Craniofacial Science; King’s College London; Population Health Research Institute; St George’s, University of London; Charles Clifford Dental Institute; Sheffield University Teaching Hospitals NHS Foundation Trust; Faculty of Dentistry, Oral & Craniofacial Science; King’s College London; London, United Kingdom;; Faculty of Dentistry; The National University of Malaysia; Faculty of Dentistry, Oral & Craniofacial Science; King’s College London; The Headache Service, Pain Management and Neuromodulation Centre; Guy’s and St. Thomas’ NHS Foundation Trust; Faculty of Dentistry, Oral & Craniofacial Science; King’s College London

**Keywords:** temporomandibular disorder, chronic widespread pain, fibromyalgia, prevalence, epidemiology, systematic review, meta-analysis

## Abstract

**Purpose:**

To evaluate the prevalence of chronic widespread pain (CWP) and fibromyalgia syndrome (FMS) in TMD patients and the prevalence of TMDs in patients with FMS.

**Method:**

A systematic search was performed in electronic databases. Studies published in English examining the prevalence of comorbid TMDs and CWP/FMS were included. The Newcastle-Ottawa Scale was used to assess study quality, and meta-analyses using defined diagnostic criteria were conducted to generate pooled prevalence estimates.

**Results:**

Nineteen studies of moderate to high quality met the selection criteria. Meta-analyses yielded a pooled prevalence rate (95% CI) for TMDs in FMS patients of 76.8% (69.5% to 83.3%). Myogenous TMDs were more prevalent in FMS patients (63.1%, 47.7% to 77.3%) than disc displacement disorders (24.2%, 19.4% to 39.5%), while a little over 40% of FMS patients had comorbid inflammatory degenerative TMDs (41.8%, 21.9% to 63.2%). Almost a third of individuals (32.7%, 4.5% to 71.0%) with TMDs had comorbid FMS, while estimates of comorbid CWP across studies ranged from 30% to 76%.

**Conclusions:**

Despite variable prevalence rates among the included studies, the present review suggests that TMDs and CWP/FMS frequently coexist, especially for individuals with painful myogenous TMDs. The clinical, pathophysiologic, and therapeutic aspects of this association are important for tailoring appropriate treatment strategies. *J Oral Facial Pain Headache 2023;37:177–193. doi: 10.11607/ofph.3260*

T*emporomandibular disorders* (TMD) is a collective term for musculoskeletal conditions affecting the temporomandibular joint (TMJ) and/or masticatory muscles. These conditions involve mandibular functional movement limitations and joint sounds,^1^ and affected individuals present with pain in the TMJ and associated anatomical structures, including symptomatic myogenous pain, arthrogenous pain, and headaches associated with TMDs.^2^ Epidemiologic studies have reported the prevalence of TMDs in different countries and found between 40% and 70% of the general population experience some signs of TMDs.^1,3–6^ These conditions occur more in women than in men, appear most often between the ages of 20 and 50, and usually peak in the fourth decade.^7,8^ TMDs are also the most common cause for seeking treatment for pain of nondental origin in the orofacial region.^9,10^ Several comorbid conditions and pathologies have been found in TMD patients, such as chronic fatigue syndrome, fibromyalgia, headache, irritable bowel syndrome, tinnitus, and depression.^11–13^

The Research Diagnostic Criteria for Temporomandibular Disorders (RDC/TMD)^14^ and the Diagnostic Criteria for Temporomandibular Disorders (DC/TMD)^15^ are commonly used for TMD diagnosis. But there are other TMD diagnostic classifications; for example, the American Association of Orofacial Pain (AAOP),^1^ the International Association for the Study of Pain (IASP),^16^ and the International Classification of Orofacial Pain (ICOP).^2^ Despite the different diagnostic criteria, the consensus is that TMDs can be subclassified into three groups: arthrogenous TMDs (including disc and joint disorders), myogenous TMDs (masticatory muscle disorders), and headache attributed to TMDs (Table 1). The diagnostic criteria for TMDs provide a comprehensive assessment and validation for each TMD categorization,^15^ whereas the IASP classification system provides very limited information for the diagnosis of TMDs. The AAOP expanded the DC/TMD taxonomy and provided evidence-based criteria for TMD diagnosis. More recently, the first International Classification of Orofacial Pain (ICOP)^2^ was developed by a collaborative group, including the AAOP, the International Headache Society (IHS), the Orofacial and Head Pain Special Interest Group (OFHP SIG) of the IASP, and the International Network for Orofacial Pain and Related Disorders Methodology (INFORM). The classification system merged the DC/TMD and pain taxonomy created by the IASP and aligned with the International Classification of Diseases (ICD-11). ICOP is a comprehensive classification system for orofacial pain conditions including painful TMDs. The classification distinguishes between muscle pain (myofascial orofacial pain) and TMJ pain, which are divided into two types: primary and secondary.

**Table 1:** Classification of TMD Subtype

TMD subtypes	TMD classification (ICOP)^1^
Myogenous	Myofascial orofacial pain Primary myofascial orofacial pain Acute primary myofascial orofacial pain Chronic primary myofascial orofacial pain Secondary myofascial orofacial pain Myofascial orofacial pain attributed to tendonitis Myofascial orofacial pain attributed to myositis Myofascial orofacial pain attributed to muscle spasm
Arthrogenous	TMJ pain Primary TMJ pain Acute primary TMJ pain Chronic primary TMJ pain Secondary TMJ pain TMJ pain attributed to arthritis TMJ pain attributed to disc displacement TMJ pain attributed to degenerative joint disease TMJ pain attributed to subluxation
Headache attributed to TMDs	Headache attributed to TMDs (ICHD-3)^2^

Fibromyalgia syndrome (FMS) is a chronic painful syndrome characterized by widespread musculoskeletal pain^17^ and is considered a subgroup of chronic widespread pain (CWP).^18^ Although the most common complaint in CWP is FMS, this condition may be related to diseases other than FMS. Several other conditions may present with CWP, including rheumatic diseases, musculoskeletal disorders, and endocrine/metabolic, neurologic, psychiatric, and medication-related conditions. Therefore, the physician should be vigilant in assessing a patient presenting with CWP.^19^ Prevalence estimates of FMS in the general population range from 0.2% to 6.6%.^19^ This disorder affects mainly individuals between the third and sixth decades of life, with a female-to-male ratio varying from 3:1 to 9:1.^20^ The diagnostic criteria for fibromyalgia have been proposed by the American College of Rheumatology (ACR) with recognition of an impaired cognitive state and somatic symptoms.^21^ Although several studies have considered the coexistence of FMS and TMDs,^22–25^ the association between these two disorders remains unexplicit. Understanding the epidemiologic perspective of this association is instrumental to appropriately diagnosing and managing patients with these conditions.

Comorbidly occurring FMS and TMD conditions can be viewed as a pair of chronic overlapping pain conditions (COPCs).^12^ COPCs are a set of disorders that coexist and include (but are not limited to) TMDs, FMS, irritable bowel syndrome (IBS), vulvodynia, chronic fatigue syndrome, interstitial cystitis, endometriosis, chronic tension-type headache, migraine headache, and chronic lower back pain. Although each condition has unique anatomical pathophysiology, they are suggestively related by common symptomatology, epidemiology, and shared underlying mechanisms. While certain psychologic features have been associated with multiple COPCs,^26^ one critical element thought to be accountable for the overlap is central sensitization, a phenomenon described by enhanced synaptic efficacy resulting in amplified sensory and nociceptive processing.^27^ The development of central sensitization or central sensitivity syndromes in FMS may contribute to multiple pain conditions, including TMJ and masticatory muscle pain.^28^

Previous systematic reviews examining the association of TMDs with FMS have reported a high prevalence of TMDs or features of TMD signs and symptoms in FMS patients^29–34^ (Table 2). For instance, Gui et al^29^ found that estimates of comorbid TMDs in FMS patients ranged across studies from 59% to 93%. However, while two recently published reviews provided weighted prevalence estimates (combing selected studies) of 14% for FMS in chronic TMDs^33,34^ and 57% for TMDs in FMS, there is a notable absence of reviews that have formally pooled prevalence data of these (coexisting) disorders using meta-analysis, and none have done so considering subtypes of TMDs. Thus, the aim of the present review was to systematically examine studies investigating comorbid CWP and/or FMS in TMD patients with masticatory muscle problems, TMJ internal derangements, and degenerative joint disease and studies assessing FMS patients with comorbid TMDs and to, where appropriate, calculate pooled prevalence estimates.

**Table 2:** Recent Systematic Reviews on the Overlap Between TMDs and FMS

Study, year	Study design	Aim	No. of studies included	Conclusions
Gui et al,^29^ 2015	Systematic review	To present a review of the literature on the relations between FMS and TMDs.	7	TMDs are highly prevalent among FMS patients, ranging from 59% to 93%. The results indicate involvement of the stomatognathic system in FMS, with myogenic disorders of the masticatory system most commonly found in those patients. FMS appears to have a series of characteristics that constitute both predisposing and triggering factors for TMDs.
Ayouni et al,^30^ 2019	Systematic review	To study the association between FMS and TMDs and the prevalence and characteristics of TMDs in patients with FMS and of FMS in patients with TMDs.	19	There was a high prevalence of TMDs in patients with FMS and a strong association between the two conditions. Muscle pain, TMJ pain, and muscle tenderness on palpation were the most common symptoms. Therefore, FMS could be an etiologic or aggravating factor for TMDs.
De Stefano et al,^31^ 2020	Systematic review	To highlight all the possible correlations between FMS and oral health.	18	There was a correlation between FMS and alterations affecting the craniomaxillofacial and craniomandibular regions. The results mainly shos an important correlation between the TMJ and the vertebral column, with all of the systemic implications arising from it.
Nascimento et al,^32^ 2020	Systematic review	To determine the prevalence of TMDs in patients with FMS.	6	The prevalence of TMDs in patients with FMS ranged from 13% to 87.1%
Kleykamp et al,^33^ 2021	Systematic review with weighted pooled prevalence	To evaluate the presence of comorbid conditions among patients with FMS.	31	The sample size–weighted (lifetime) prevalence of comorbid TMDs is 57%. The prevalence of other comorbid chronic pain conditions (ie, chronic tension-type or migraine headache, IBS) was also high, ranging from 39% to 76%. Lifetime prevalence of comorbid depression/major depressive disorder in FMS was 63%, while almost one-third of FMS patients had current or lifetime bipolar disorder, panic disorder, or posttraumatic stress disorder.
Kleykamp et al,^34^ 2022	Systematic review with weighted pooled prevalence	To evaluate the presence of comorbid conditions among patients with TMDs.	9	The sample size–weighted prevalence of comorbid FMS is 14%. There is a high prevalence of other (comorbid) chronic pain conditions among patients with TMDs: current chronic back pain (66%), myofascial syndrome (50%), chronic stomach pain (50%), chronic migraine headache (40%), and IBS (19%). Psychiatric disorders among patients with different types of TMDs were studied less commonly in this pain population.

## Materials and Methods

The search strategy and protocol were registered and are available in the PROSPERO database.^35^ The PICO model^36^ and PRISMA guidelines^37^ were used for data synthesis and reporting with meta-analysis.

### Search Strategy

The search was conducted in the following electronic bibliographic databases: PubMed, CINAHL, Web of Science, MEDLINE, PsycINFO, Scopus, Embase, and EBM Review Cochrane (published up to April 2020). Additional literature searches in Google Scholar, OpenGrey, and the reference lists of downloaded articles were also performed. We used search keywords for TMDs with the following terms: temporomandibular disorder, jaw joint pain, orofacial pain, facial pain, myofascial, aching jaw, mandibular dysfunction, masticatory system disorder, and oro-mandibular disorder; and combined with “AND” followed by fibromyalgia terms: fibromyalgia, and chronic widespread pain. The search was conducted during April to May 2020.

### Eligibility

Inclusion criteria followed the PECOS process (population, exposure, comparison, outcome, study design). The review considered studies with no restriction of participants regarding age, sex, or other characteristics (population). Informal and formal standardized diagnostic or defined clinician-based criteria were described in included studies (exposure, comparison); for example, in TMDs (painful or nonpainful), formal diagnosis was made using the RDC/TMD, DC/TMD, and AAOP criteria, and for FMS, the ACR criteria were used. Studies included one or more measurements of prevalence rates of TMDs in CWP/FMS and/or of CWP/FMS in TMDs (outcome). Observational study designs, such as prospective, case-control, cohort, and cross-sectional, were included (study design). All retrieved articles are accessible, published in the English language, and without time limitations.

### Data Extraction and Analysis

Studies were selected on the basis of the previously mentioned criteria and the presence of the proportion of TMD patients with comorbid FMS and/or vice versa. Association measurements between the TMD group and FMS group (prevalence rate, odds ratio) were additionally collected. One reviewer (P.Y.) screened initially, then four reviewers (P.Y., J.S., P.C., P.R.) independently assessed full articles for inclusion in the reviews. In case of difficulties and disagreements, the reviewers discussed and resolved before achieving consensus. The following information was extracted from the included studies: author and year of publication, study design, sample size and source of the sample, location of study, sample demographics, method of diagnosis of TMDs and FMS, and outcomes.

A meta-analysis was undertaken by pooling the prevalence rates from relevant studies. Meta-analyses included only studies with adult populations whereby formal diagnosis of FMS had been made according to ACR criteria (determined via clinical evaluation or previous diagnosis) and formal diagnosis of TMDs had been made via clinical evaluation according to standardized criteria such as the RDC/TMD or AAOP, or via clinical assessments and/or use of structural questionnaires guided by diagnostic criteria. Studies in which FMS and/or TMDs were ascribed via self-report of symptoms or symptom history or by examination of TMD signs were excluded from data pooling. Similarly, as CWP was typically determined via participant self-report in the included studies (and the criteria varied greatly), it was not possible to perform a meta-analysis of prevalence rates relating to CWP. Fixed- or random-effects meta-analyses were conducted using Freeman-Tukey transformations to calculate weighted summary proportions.^38^ Prevalence estimates were presented with 95% CI, and Cochran Q and I^2^ statistics were calculated to indicate the presence of heterogeneity. Random-effects modeling was applied where there was high heterogeneity across included studies (I^2^ > 50%).^39^ Forest plots were created for all estimates. Analyses were performed using SPSS (version 26.0, IBM) and MedCalc Statistical Software.

### Study Quality and Weight of Evidence

Rating for study quality was assessed using the Newcastle-Ottawa Scale.^40^ The checklist for quality criteria is shown in Appendix Table 1. The scoring of all criteria is based on the Newcastle-Ottawa guideline. The measure of sample size was adjusted by rating one star if the number of participants was ≥ 100 per group, which we considered an appropriate number for representative samples. To specifically analyze each study’s appropriateness with respect to the review question on prevalence, we additionally appraised each article based on Gough’s Weight of Evidence Framework (WoE),^41^ with the aim to evaluate, in particular, whether an individual study was suitable to answer the review question rather than the study question in general. The study quality and WoE were performed by the same group of reviewers.

## Results

### Search and Review Results

**Fig 1:**
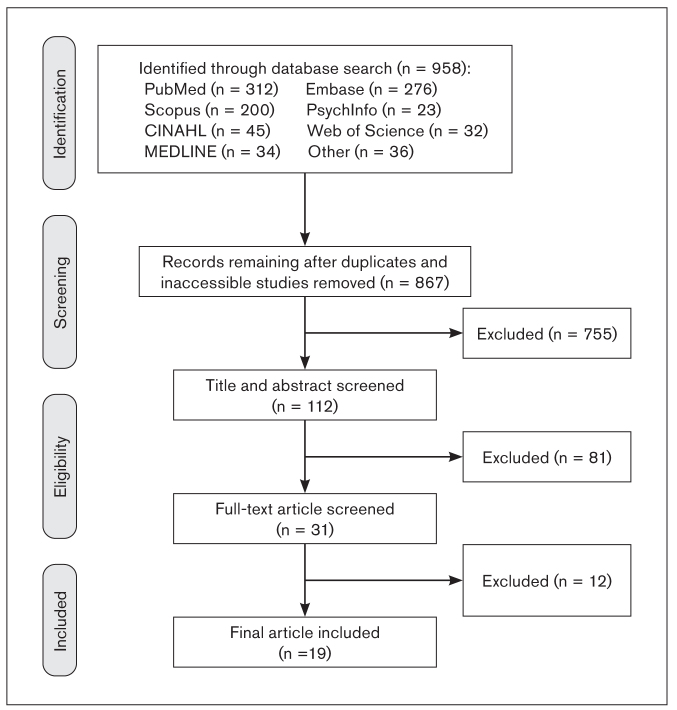
Flowchart showing study selection process.

The results from the database search is shown in Fig 1. All titles and abstracts were initially screened, and eligible articles were investigated further according to the eligibility criteria and their relevance to the review question. This systematic review included 19 studies in total. Studies were grouped into two data groups: the prevalence of CWP/FMS in people with TMDs (9 studies), and the prevalence of TMDs in people with CWP/FMS (10 studies). We present characteristics of studies individually by research design, country of survey, number, source, sex, and mean age of participants, diagnostic criteria and method used for FMS/CWP and TMD, and the prevalence found. Tables 3^11,42–49^ and 4^22–25,50–55^ describe the characteristics and results of the selected investigations of CWP/FMS prevalence in TMD groups and TMD prevalence in FMS groups, respectively.

**Table 3:** Characteristics of Included studies on Prevalence of CWP and FMS in TMD patients (n = 9)

Study design		Study, year	Location	Sample size and composition, sex (% female) and age	Source of the sample	TMD diagnosis	FMS diagnosis	Main outcome (prevalence of CWP or FMS)	Additional outcome(s)
Case-control	1	Aaron et al,^42^ 2000	USA	50 patients; 25 FMS patients, TMD 25 patients (all TMD subtypes): F = 73% to 96% (across groups), mean age FMS = 48.5 y, TMD = 38.0 y 22 controls (age data not provided)	University hospitals	Clinical assessment using the RDC/TMD	Clinical evaluation according to ACR criteria	FMS = 13%	–
	2	Hoffman et al,^11^ 2011	USA	1,511 TMD patients (all TMD subtypes): F = 90%, mean age = 41 y 57 controls: a 1-to-4 control-to-subject match based on age, sex, and education	Web-based registry of the TMJ Association Unaffected TMD friends	Self-reported TMD symptoms	Self-reported diagnosis	FMS = 18%	Before onset of TMD, 4% of the TMD samples reported FMS. After TMD onset, 21% of them experienced FMS (*P* < .001).
	3	de Siqueira et al,^43^ 2013	Brazil	75 orofacial pain patients, of which FMS = 8, TMD = 11 (all TMD subtypes): FMS = 100% F, TMD = 100% F, mean ± SD age: FM = 47.0 ± 1.2 y, TMD = 43.6 ± 17.8 y 41 controls: F = 46.3%, mean age ± SD = 63.9 ± 20.3 y	Orofacial pain clinic Preventive medical clinics	Clinical evaluation according to IHS criteria	Previous diagnosis based on ACR criteria; generalized pain complaints (CWP)	TMD: generalized pain = 72.7%	–
Cross-sectional	4	Wright et al,^44^ 1997	USA	104 TMD patients (all TMD subtypes): F = 81.7%, mean age = 33 y (range 18–76)	TMD specialty clinic	Questions and clinical examination as described by RDC/TMD	Questions related to history of symptoms	FMS = 20%	–
	5	Raphael et al,^45^ 2000	USA	162 myofascial pain patients: F = 100%, age range 18–65 y	Medical records from orofacial pain specialist	Criteria established by the IASP	Patient self-report	FMS = 23.5%	Onset of FMS and TMD most often occurred within the same year. If they did not, the facial pain most often preceded the widespread pain.
	6	Leblebici et al,^46^ 2007	Turkey	52 patients; 31 FMS patients with possible TMD, 21 TMD patients (myofascial pain with/without arthrogenous origin) with possible FMS: F = 100%; mean ± SD age = 35.2 ± 10.2 y	University hospitals	Clinical assessment with explained criteria		Clinic evaluation based on ACR criteria	FMS = 52% in TMD patients; TMD = 80% in FMS patients.
Cohort	7	John et al,^47^ 2003	USA	397 TMD patients (painful dysfunctional TMD): F = 82.6%, age range = 18–74 y	TMD clinics of Group Health Cooperative	Patient history of previous diagnosis of TMD (at least 1 y of seeing physician or dentists in TMD clinics)	Questions referred to the previous 6-mo period of widespread pain (CWP)	Generalized pain = 75.57%	Among samples without dysfunctional TMD pain at baseline, widespread pain was a risk factor for development of dysfunctional TMD pain (OR = 1.9, 95% CI = 1.2–2.8, *P* = .003).
	8	Velly et al,^48^ 2010	USA	572 TMD patients, I: 262 in onset cohort (clinically nonsignificant pain), II: 310 in persistence cohort with (clinically significant pain): I: F = 84%, II: F = 95%; mean ± SD age: I = 36.3 ± 12.5 y; II = 35.6 ± 12.5 y	General populations from 2 areas in community	Clinical evaluation according to Craniomandibular Index redesigned to conform with RDC/TMD–CMI/RDC	Clinical assessment based on the ACR criteria; question about experiencing widespread bodily pain (on both right and left sides, above and below waist) (CWP)	FMS = 11%; CWP = 30.6%	Persistence of clinically significant pain was related to FMS and depression.
	9	Losert-Bruggner et al,^49^ 2017	Germany	555 samples with pain from CMD and/or CCD: F = 64.1%; age data not provided	Patient record with diagnosis of CCD or CMD > 6 mo	Clinical evaluation according to RDC/TMD	Clinical investigation using S3 guidelines of the AWMF 2012)* and modified ACR	FMS = 63%	The mean pain intensity of patients with FMS was 8.3 (scale 1–10), whereas patients without FMS had a mean pain intensity of 5.5 (*P* < .01).

AWMF = Arbeitsgemeinschaft der Wissenschafylichen Medizinischen Fachgesellschaften (Association of the Medical Scientific Societies of Germany); CCD = craniocervical dysfunction; CMD = craniomandibular dysfunction; CWP = chronic widespread pain; MMP = masticatory myofascial pain.

**Table 4:** Characteristics of Included studies on Prevalence of TMD in FMS Patients (n = 10)

Study design		Study, year	Location	Sample size and composition, sex (% female) and age	Source of the sample	TMD diagnosis	FMS diagnosis	Main outcome (Prevalence of CWP or FMS)	Additional outcome
Case-control	1	Rhodus et al,^50^ 2003	USA	67 FMS, F = 100%; mean ± SD age = 47.6 ± 2.3 y 67 controls, F = 100%, age data not provided	Rheumatology Clinic Volunteers (matched by age and sex)	Rheumatic Problems Questionnaire developed from university TMJ Clinic	Clinical assessment based on ACR criteria	TMD = 67.6% in FMS group TMD = 20.0% in control group (*P* < .001)	–
	2	Balasubramaniam et al,^51^2007	USA	32 FMS, F = 100%; mean ± SD age = 52.2 ± 7.8 y 19 FBS as controls, F = 68.4%, mean ± SD age = 50.0 ± 9.1 y	Patients from Physical Medicine and Rehabilitation Clinic and an FMS workshop	Questionnaire and clinic evaluation guided by RDC/TMD	Clinical assessment based on ACR criteria	TMD = 59.4% in FMS group: muscle disorders = 43.8%, disc displacement = 12.5%, inflammatory-degenerative disorders = 46.9% TMD = 15.8% in control (FBS) group	Among the FMS group, the patients presenting facial pain were not significantly more likely to meet the RDC/TMD criteria compared to the patients without facial pain (*P* = .17, OR = 2.74, 95% CI = 0.64–11.75).
	3	Salvetti et al,^25^ 2007	Italy	93 FMS, F = 94.6%, mean ± SD age = 50.1 ± 9.8 y 181 TMD as controls, F = 75.7%; mean ± SD age = 40.7 ± 7.4 y	Rheumatology Disease Department Section of Prosthetic Dentistry	Clinic evaluation according to RDC/TMD	Previous diagnosis based on ACR criteria	TMD = 79.6%: muscle disorders = 40.9%, disc displacement = 29.0%, inflammatory-degenerative disorders = 71.0%	–
	4	Silva et al,^23^ 2012	Brazil	25 FMS, F = 96.0%; mean ± SD age = 47.7 ± 9.9 y 67 controls, F = 96.0%, mean ± SD age = 52.2 ± 17.6 y	Division of Trauma and Orthopedic Institute Unspecified	Clinic assessment according to AAOP	Previous diagnosis based on ACR criteria	TMD = 88.0% in FMS group TMD = 20.0% in control group (*P* < .001)	The FMS group reported more fatigue complaints in the orofacial region (*P* = .002) and a higher number of painful areas upon palpation of the head and neck (*P* = .001) than the healthy control group.
	5	Pimentel et al,^24^ 2013	Brazil	40 FMS, F = 100.0%, mean ± SD age = 53.5 ± 9.2 y 40 healthy controls, F = 100.0%; mean ± SD age = 51.5 ± 11.5 y	Hospital rheumatologist Dental college	Clinic evaluation according to RDC/TMD	Clinic evaluation based on ACR criteria	FMS group: myofascial pain = 77.5%, disc displacement = 22.5%, inflammatory-degenerative joint disorders = 42.5%, control group: myofascial pain = 10.0%, disc displacement = 30.0%, inflammatory-degenerative joint disorders = 35.0%	FM patients were significantly more likely to have facial muscle pain than patients without FMS (*P* < .001; OR = 31.0, 95% CI = 8.6–110.6).
	6	García-Moya et al,^52^ 2015	Spain	20 FMS, F = 100.0%, age range = 35–60 y 18 controls, F = 100.0%, age range = 35–60 y	Fibromyalgia association Different dental practices	Self-report according to AAOP, TMD signs assessment	Previous diagnosis based on ACR criteria	FMS patients reported more TMD signs and symptoms than controls, as well as pain or difficulty in opening the mouth (60% vs 22.2%), pain or difficulty in speaking or chewing (60% vs 22.2%), and pain in ears, temples, or cheeks (95% vs. 44%)	100% of FM patients presented at least three affirmative answers (yes to checklist questionnaires) compared to 50% of the control group.
	7	Zwir et al,^53^ 2018	Brazil	12 FMS, F = 80.0%, mean age = 13.1 y (range = 6–18) 20 controls, F = 80.0%, mean age = 12.8 y (range = 6–18)	Pediatric rheumatology division	Questionnaire and clinic examination (defined criteria)	Clinic evaluation based on ACR criteria	TMD symptoms = 75% in FMS group and 15% in control 15% (*P* = .001)	–
Cross-sectional	8	Fraga et al,^22^ 2012	Brazil	60 FMS, F = 86.7%, mean ± SD age = 49.2 ± 13.8 y	University hospitals	Clinical assessment according to RDC/TMD	Previous confirmed diagnosis guided by ACR criteria	Myofascial pain = 61.67%; disc displacement with reduction = 1.7%, disc displacement without reduction = 21.6%, osteoarthritis = 36.7%, arthralgia = 28.3%, osteoarthrosis = 1.7%	93.3% reported tenderness in the masticatory muscles (at least 1 muscle), and 83.3% reported TMJ pain.
	9	Gui et al,^54^ 2013	Brazil	41 FMS, F = 100.0%, mean ± SD age = 53.2 ± 5.6 y	University hospitals	Clinical assessment according to RDC/TMD	Clinic evaluation based on ACR criteria	TMD = 87.1%; myofascial pain = 87.1%, disc displacement disorder = 12.9%; inflammatory joint disease =16.1%	–
	10	Di Venere et al,^55^ 2015	Italy	31 FMS, F = 90.3%, mean ± SD age = 47.9 ± 9.9 y	University hospitals	Clinical assessment according to RDC/TMD	Previous confirmed diagnosis guided by ACR criteria	Symptoms and signs of craniomandibular disorders = 80.6%; myofascial pain = 67.7%; disc displacement disorder = 35.5%, inflammatory joint disease = 9.7%	–

FBS = failed back syndrome.

In summary, of the 19 studies retrieved, 10 were case-control design,^11,23–25,42,43,50–53^ 6 were cross-sectional,^22,44–46,54,55^ and 3 were cohort studies.^47–49^ Two of the included studies were population-based samples, while the remainder (17 studies) used clinic-based sampling. The sex breakdown of study samples varied considerably, with some studies examining women only.^24,43,45,46,50,52,54^ Most of the studies were conducted in adults (aged 18 to 75 years), except for one study of Brazilian adolescents (aged 12 to 13 years).^53^ TMDs and FMS assessment were conducted in different ways across retrieved studies: for TMDs, 2 studies relied on self-reports, 4 studies used questionnaires, and 13 studies performed formal clinical examinations; for FMS, 2 studies relied on self-reports, 2 used questionnaires, and 15 performed formal clinical evaluations. Meta-analyses were subsequently administered by pooling studies according to the eligibility criteria described above.

Quality assessment of included studies was scored and shown in Table 5 for FMS in TMD patients and Table 6 for TMDs in FMS patients, while the WoE is reported in Table 7. All included studies achieved ≥ 5 stars (out of 9 stars), which represents moderate to high quality. Most articles obtained moderate to high WoE, indicating that the included articles are suitable to answer our question about the prevalence of TMDs or FMS.

**Table 5:** Quality Assessment (Newcastle-Ottawa Scale) of Studies on Prevalence of CWP/FMS in TMD Patients (n = 9)

Studies	Selection				Total (9*)
**Case-control**	Adequate case definition (*)	Representativeness of the cases (*)	Selection of controls (*)	Definition of controls (*)	
Aaron et al^42^	*			*	5
Hoffmann et al^11^		*	*	*	5
de Siqueira et al^43^	*		*	*	6
**Cross-sectional**	Representativeness of the sample (*)	Sample size (*)	Nonrespondents (*)	Validated measurement tool (**)	Total (9*)
Wright et al^44^	*	*		*	6
Raphael et al^45^	*	*		*	5
Leblebici et al^46^	*			**	7
**Cohort**	Representativeness of the exposed cohort (*)	Selection of the nonexposed cohort (*)	Ascertainment of exposure (*)	No outcome of interest was presented (*)	Total (9*)
John et al^47^	*			*	6
Velly et al^48^	*	*	*	*	9
Losert-Bruggner et al^49^	*		*	*	6

Studies	Compatibility	Outcome			Total (9*)
**Case-control**	Comparability of cases and controls (**)	Ascertainment of exposure (*)	Same method of ascertainment (*)	Nonresponse rate (*)	
Aaron et al^42^	**		*		5
Hoffmann et al^11^	*		*		5
de Siqueira et al^43^	*	*	*		6
**Cross-sectional**	Comparable subjects, controlled confounding (*)	Assessment of outcome (**)	Appropriate statistics used (*)		Total (9*)
Wright et al^44^		**	*		6
Raphael et al^45^		*	*		5
Leblebici et al^46^	*	**	*		7
**Cohort**	Comparability of the design, analysis (**)	Assessment of outcome (*)	Follow-up long enough (*)	Adequacy of follow-up of cohorts (*)	Total (9*)
John et al^47^	*	*	*	*	6
Velly et al^48^	**	*	*	*	9
Losert-Bruggner et al^49^	*	*		*	6

Rating guidelines are shown in Appendix Table 1.

**Table 6:** Quality Assessment (Newcastle-Ottawa Scale) of Studies on Prevalence of TMD in FMS Patients (n = 10)

Studies	Selection				Total (9*)
**Case-control**	Adequate case definition (*)	Representativeness of the cases (*)	Selection of controls (*)	Definition of controls (*)	
Rhodus et al^50^	*		*	*	7
Balasubramaniam et al^51^	*			*	6
Salvetti et al^25^	*	*	*	*	7
da Silva et al^23^	*		*	*	7
Pimentel et al^24^	*			*	5
García-Moya et al^52^	*			*	6
Zwir et al^53^	*			*	5
**Cross-sectional**	Representativeness of the sample (*)	Sample size (*)	Nonrespondents (*)	Validated measurement tool (**)	Total (9*)
Fraga et al^22^	*			**	6
Gui et al^54^	*			**	6
Di Venere et al^55^	*			**	6

Studies	Compatibility	Outcome			Total (9*)
**Case-control**	Comparability of the design, analysis (**)	Ascertainment of exposure (*)	Same method of ascertainment (*)	Nonresponse rate (*)	
Rhodus et al^50^	**	*	*		7
Balasubramaniam et al^51^	**	*	*		6
Salvetti et al^25^	*	*	*		7
da Silva et al^23^	**	*	*		7
Pimentel et al^24^	*	*	*		5
García-Moya et al^52^	*	*	*		6
Zwir et al^53^	*	*	*		5
**Cross-sectional**	Comparable subjects, controlled confounding (*)	Assessment of outcome (**)	Appropriate statistics used (*)		Total (9*)
Fraga et al^22^	*	**			6
Gui et al^54^	*	*	*		6
Di Venere et al^55^	*	*	*		6

Rating guidelines are shown in Appendix Table 1.

**Table 7:** Weight of Evidence Ratings for Outcome Studies

Study	Overall soundness of study in answering the study question	Appropriateness of research methodology	Relevance of focus for addressing systematic review question	Overall rating
**Studies on FMS prevalence in TMDs**				
Wright et al^44^	Medium	Medium	Medium	Medium
Aaron et al^42^	Medium	High	Medium	Medium
Raphael et al^45^	Medium	Medium	Medium	Medium
John et al^47^	Medium	Medium	Medium	Medium
Leblebici et al^46^	High	High	Medium	High
Velly et al^48^	Medium	High	High	High
Hoffmann et al^11^	Medium	Medium	Medium	Medium
de Siqueira et al^43^	Medium	Medium	Medium	Medium
Losert-Bruggner et al^49^	Medium	High	Medium	Medium
**Studies on TMD prevalence in FMS**				
Rhodus et al^50^	High	Medium	Medium	Medium
Balasubramaniam et al^51^	Medium	High	Medium	Medium
Salvetti et al^25^	High	Medium	Medium	Medium
Fraga et al^22^	Medium	Medium	Medium	Medium
da Silva et al^23^	High	Medium	Medium	Medium
Gui et al^54^	Medium	High	Medium	Medium
Pimentel et al^24^	Medium	High	Medium	Medium
García-Moya et al^52^	Medium	Medium	Medium	Medium
Di Venere et al^55^	Medium	Medium	Medium	Medium
Zwir et al^53^	Medium	High	Medium	Medium

### Prevalence of CWP in Patients Experiencing TMDs

Three studies^43,47,48^ considered the prevalence of CWP in a TMD population. The approach to classifying patients with widespread pain varied markedly across these studies, precluding the possibility of formal data pooling. Two studies with clinical samples reported prevalence estimates greater than 70% (72.7% and 75.6%).^43,47^ Both studies, however, adopted generous criteria to determine widespread pain (≥ 1 body pain sites in last 6 months or “generalized pain complaints”). Velly et al^48^ employed more conservative criteria (a “yes” response to questionnaire item “Do you experience widespread bodily pain [on both your right and left sides as well as above and below the waist]?” in a community-based sample and reported a modest prevalence rate of 30.6%.

### Prevalence of FMS in Patients Experiencing TMDs

The estimated pooled proportion (95% CI) of FMS in TMD patients reported from four studies^42,46,48,49^ was 32.7% (4.5% to 71.0%; Appendix Fig 1). Large heterogeneity was observed across studies (I^2^ > 99.3%; *P* < .001). Studies used different clinical assessment criteria and varying TMD populations (eg, nonpainful/painful TMD). The highest proportions were in a study of patients with painful disorders of the masticatory muscles and TMJs lasting beyond 6 months (63.2%, 59.1% to 67.3%) and from a smaller clinical study of TMD patients referred to a physiatrist for the evaluation of possible FMS (52.4%, 29.8% to 74.3%), for which prevalence was especially high in patients with masticatory myofascial pain (9/11 or 81.8%). In contrast, the lowest proportion was derived from a population-based study of people with TMDs that included those with clinically nonsignificant TMD pain (10.5%, 8.1% to 13.3%).

### Prevalence of TMDs in Patients Experiencing FMS

The pooled prevalence estimate (95% CI) for TMDs in people experiencing FMS^23,25,46,50,51,54,55^ was 76.8% (69.5% to 83.3%; Appendix Fig 2). Medium heterogeneity among seven studies was found (I^2^ = 51.1%). The two lowest prevalence estimates were observed in studies where TMD was determined via clinical examination guided by diagnostic criteria or responses to structured questionnaire items derived from diagnostic criteria (rather than formal clinical diagnostic assessment). When data pooling was done without these studies, the pooled prevalence estimate increased slightly to 81.4% (75.5% to 86.3%) with little evidence for cross-study heterogeneity (Q(6) = 1.4; *P* = .842; I^2^ = 0.0%).

A subgroup meta-analysis was additionally conducted according to TMD subtype: muscle, disc displacement, and inflammatory-degenerative disorders (Appendix Fig 3). Pooled estimates (95% CI) indicated more than 60% (63.0%, 47.7% to 77.3%) of FMS patients presented with myogenous TMDs, while 41.8% (21.9% to 63.2%) presented with inflammatory degenerative disorders. Almost a quarter (24.2%, 19.4% to 39.5%) of FMS patients also had disc displacement disorder (Appendix Fig 3b). Studies concerning the prevalence of muscle and inflammatory degenerative disorders in FMS were notably heterogenous (I^2^ > 85%; *P* < .001), but less heterogeneity was found across studies of comorbid disc displacement disorder (I^2^ = 37.6%). Only one study in each subgroup meta-analysis relied on clinical examination guided by diagnostic criteria (rather than formal diagnosis) to determine TMD subtype classification. When this study was excluded from analyses, prevalence estimates remained largely the same (myogenous TMDs = 66.7%, 49.3% to 81.9%; disc displacement disorder = 25.7%, 20.5% to 31.5%; and inflammatory-degenerative disorders = 40.7%, 17.8% to 66.0%) and did not serve to decrease high levels of heterogeneity in muscle (I^2^ = 87.3%) and inflammatory-degenerative disorders (I^2^ = 94.0%).

## Discussion

The current systematic review included a total of 19 articles—9 studies on the prevalence of CWP or FMS in TMD patients, and 10 studies on the proportion of TMDs in FMS patients.

As noted in previous reviews in this area,^32,34^ the various criteria guidelines or protocols used in diagnosing TMDs, CWP, and FMS and their subjective dependence on patient symptoms and clinician assessment, as well as their differences in application over time (eg, ACR criteria revisions), contribute to the heterogeneity of the pooled studies. More specifically, the various classification systems for TMDs create a field of diagnostic confusion. There is uncertainty, overlap, and many different terminologies that refer to similar entities. A unified consensus would minimize confusion for physicians and patients. When clinicians use the same criteria and taxonomy, clinical questions and experiences can be more easily translated into relevant research questions.

Four studies included measurements of the prevalence of FMS in TMDs.^42,46,48,49^ Although there is large heterogeneity in the results of the meta-analysis, the observed pooled prevalence (32%) supports the hypothesis of increased risk of (comorbid) FMS in people experiencing (painful) TMDs. Heterogeneity across studies likely reflects differences in sample composition. Participants with TMDs in Velly et al^48^ were recruited from the community and did not present with painful TMDs. Conversely, patients in the clinical studies of Leblebici et al^46^ and Losert-Bruggner et al^49^ were seeking or undergoing treatment to alleviate persistent painful TMD symptoms. The distinction is likely to be important with respect to rates of comorbid FMS (or CWP) and accounting for discrepancies with the lower (pooled) prevalence of 14% reported in the recent review performed by Kleykamp et al.^34^ For example, Nguyen et al found that coexisting pain beyond orofacial areas (eg, pain in the neck or abdomen) was more frequently observed in patients with chronic TMD pain compared to acute TMD symptoms.^56^ A recent study using voxel-based morphometry reported that, relative to controls, TMD patients drawn from clinic-based samples showed smaller gray matter volume in the anterior medial cingulate cortex reaching into the medial prefrontal cortex (a marker of vulnerability to CPS development), whereas no significant differences between controls and participants with TMD symptoms recruited from the community were observed.^57^

Three studies^34,38,39^ explored widespread bodily pain (CWP) in TMD populations; prevalence estimates in individual studies tended to be higher than in studies of co-occurring TMD and FMS, a likely consequence of the assessed CWP populations falling below the threshold of an FMS diagnosis. Patients are often preliminarily diagnosed with CWP before receiving a diagnosis of FMS by exclusion of other possible contributing conditions, such as inflammatory rheumatic diseases, nonrheumatic musculoskeletal conditions (hypermobile joints), nonrheumatic medical conditions (infections, malignancy, thyroid disease), neurologic diseases (Parkinson disease), spinal stenosis, myopathy, mental health disorders, and medication-induced pain conditions (opioids, chemotherapy).^58^ Aside from the sample differences noted above, the discrepancy in rates across included studies probably reflects divergent CWP classification methods. One study with a lower prevalence^48^ asked a specific question about experiencing widespread bodily pain on both the right and left sides, as well as above and below the waist, to assess CWP. The other two studies that observed > 70% CWP comorbidity used less conservative criteria (≥ 1 body pain site[s] in the past 6 months or “generalized pain complaints”).^43,47^ Although further work is needed using established widespread pain criteria to better quantify the TMD and CWP association, it appears that a not insignificant number of TMD patients present with pain outside the orofacial region. To the extent that TMD patients with widespread pain present with more psychologic distress and respond less favorably to conventional TMD treatment,^59^ routine consideration of the presence of widespread bodily pain is an important indicator of treatment strategy with a view to prevent an increase in the number of pain sites and severity of pain at affected sites.

In line with previous reviews concerning the association between TMDs and FMS,^29,30^ the estimated pooled prevalence from this meta-analysis suggested that three-quarters (76%) of patients with FMS have TMDs and that studies were largely consistent. Analysis on TMD subtype revealed that FMS patients more commonly presented with a myogenic disorder of the masticatory system (63%) than inflammatory-degenerative disorders of the TMJ (42%) or disc displacement disorders (24%), although some pooled estimates had wide CIs (because of the moderate to high heterogeneity), and only the CIs of prevalence rates for myogenic disorders of the masticatory systems and disc displacement orders did not overlap. Nevertheless, this finding coincided with a previous study suggesting that TMD signs reported by FMS patients were most often tenderness of the masticatory muscles (93.3%) and the TMJ (83.3%), while a smaller percentage of FMS patients had joint sounds (63.3%).^22^

Both TMDs and FMS are COPCs that share similar inflammatory and hyperalgesic features or symptoms of the facial and cervical musculoskeletal structures^24,60^ but are nevertheless discrete conditions. FMS patients have a lower pain threshold, frequently experience fatigue, and have a lower muscle burden than TMD patients. However, FMS groups show a high prevalence of TMDs and pain in sites upon palpation of the head and neck area, frequently complain of fatigue in the orofacial region, and experience pain with jaw movements.^23^ There are no clear etiologies or pathogenesis on the development of coexisting TMDs and FMS. Multiple risk factors such as trauma, oral-facial parafunctional habits, and connective tissue diseases contribute to TMD development, while FMS is related to dysfunction of the central nervous system, genetics, and hormone and metabolite imbalance.^61^ There is a high frequency of psychophysiologic and psychiatric disorders in patients with TMDs and patients with FMS, including sleep disturbances, depressive and anxiety disorders, oral ulcers, and other COPCs.^33,34,61^ Two recent reviews indicated that the prevalence of other COPCs (eg, chronic back pain, chronic stomach pain, chronic migraine headache, and IBS) ranged from 39% to 76% in FMS patients and from 19% to 66% in individuals with TMDs depending on the specific condition.^33,34^ These coexisting conditions contribute to the complexity of TMDs and likely increase the overall pain burden associated with this group of conditions.

Our review suggests that about one-third of people with TMDs have comorbid FMS, although there is wide variation according to sample composition, whereas more than three-quarters of the FMS population have comorbid TMDs, with much less variation in prevalence across the latter set of studies. The higher prevalence of TMDs in FMS than of FMS in TMDs is consistent with some previous studies^62,63^ and likely reflects the neuromuscular impairment and central sensitization that characterizes FMS, which could lead to the temporomandibular musculoskeletal system’s failure to adapt to continuous stress and loading.^64^ Furthermore, the overlapping neuroinflammatory pathophysiology in TMDs and CWP leads to the argument that the subgroup of hypersensitive TMD patients could transit to CWP.^64^ This is supported by evidence that individuals with signs of painful TMDs are at higher risk of developing central sensitization than pain-free adolescents.^65^

The precise relationship between timing of onset of coexisting TMDs and FMS remains unclear. One study^45^ suggested that TMDs and FMS frequently occurred within the same year; if not, facial pain preceded widespread pain in most patients. Hoffmann et al^11^ also reported that 4% of TMD patients experienced FMS before developing TMDs, and the proportion of FMS increased significantly to 21% after the onset of TMDs. In addition, other studies have suggested that CWP and FMS had a partial influence on the occurrence of clinically significant TMD pain and its persistence.^48^ John et al reported that CWP predicted the occurrence of dysfunctional TMD pain in women (but not in men) as well as its persistence.^47^ Furthermore, previous studies have found that the presence of multiple painful areas elsewhere in the body may increase the risk of onset of TMD pain within the next 3 years^66,67^ and that the level of facial pain is positively correlated with that of general body pain.^68^

While research findings concerning whether the emergence of TMDs precedes, coincides, or follows the development of CWP or FMS remains inconsistent, the evidence for an elevated risk of overlapping conditions firmly indicates clinicians should be wary of the possible coexistence and exacerbation of TMD pain when CWP or FMS has developed. From this view, investigation of other (bodily) clinical pain features reported by the patient should better enable more comprehensive TMD patient management. Harper et al^69^ recently reported that higher levels of FMS symptoms were associated with greater pain at rest and higher perceived functional limitation of the jaw in TMD patients, suggesting that treatments aimed at decreasing central pain sensitization and reducing spontaneous pain may also contribute to TMD symptom relief. More generally, combined management of the contributing factors to TMDs and FMS may improve patients’ oral and general quality of life, with pain reduction and improved temporomandibular system physical functions.^70^

### Limitations

This review included a variety of study designs, involving those with and without control groups, but the difference in prevalence estimates between patient and control samples with respect to comorbid conditions were not examined in the meta-analysis. In addition, our systematic review included only English-language publications. Thus, further reviews without language restriction will obtain more data and reduce systematic bias. As noted, data from this review were pooled across studies that used diagnostic criteria that differed according to classification systems and revisions over time, which can lead to disparity in prevalence rates.^59^ The recently published ICOP-2, endorsed by most of the leading orofacial pain institutions, will help to reduce diagnostic ambiguity and improve consistency across TMD studies in future reviews. Study samples often had a female dominance, likely reflecting that, for example, 80% to 90% of populations with the FMS condition are female.^71^ But this potentially limits the representativeness of the FMS and CWP populations included in the review. Further, the TMD samples in most studies were individuals with painful TMDs who sought treatment, presumably to relieve TMD pain. However, TMDs are a collective condition associated with pain and/or dysfunction, and therefore the inclusion of individuals with painful TMDs does not reflect the broader population with TMDs and these patients may be more vulnerable to the development of chronic widespread pain or FMS. Accordingly, analyses investigating the pooled prevalence rate of FMS in people with TMDs incorporated studies with both clinical and population-based samples, yielding large heterogeneity in outcomes. While subgroup analyses based on sample composition could be considered, it is of limited value when the number of studies included in the meta-analyses is too small to perform meaningful analyses. In any case, some caution is warranted when interpreting these findings.

Finally, this systematic review was conducted in 2020. By the time of publication, there were additional empirical studies examining the coexistence of TMDs and FMS/CWP, although these tended to reaffirm findings from the present review. For instance, a recent Swedish study reported that almost 30% (47 of 161) of patients referred to orofacial pain clinics fulfilled the ACR (2016) criteria for FMS, although the rate was much higher in the subgroup of patients with myofascial pain with referral (45.7%) than those with myalgia (12.5%).^72^ In a US study of clinic patients who presented for treatment, 17 of 89 (19.1%) patients with TMDs screened positive for FMS.^69^ Another recent Swedish study, but with a community-based sample, found the overlap between widespread pain (≥ 7 pain sites identified from a full-body pain diagram) and any myofascial orofacial pain diagnosis was 57.3%.^73^ Altogether, these recent studies support the findings of this review, suggesting that individuals with TMDs, particularly those seeking treatment for painful myogenous TMDs, have an elevated risk of experiencing concurrent widespread pain and/or having FMS.

## Conclusions

This systematic review found a high co-occurrence of TMDs and FMS. Pooled prevalence estimates indicate that about one-third of TMD patients have FMS, whereas more than three-quarters of the FMS population have comorbid TMDs with higher rates of myogenous TMDs than disc displacement disorders. The variability in TMD sample composition across studies investigating comorbid FMS yielded marked heterogeneity in the corresponding meta-analysis, complicating the interpretation of overall prevalence. Experience of CWP was also common in people with TMDs, with estimates across individual studies ranging from 30% to 76%, although the criteria used to classify CWP varied greatly. These findings suggest a need for clinicians to consider the overlap between TMDs and CWP/FMS when treating affected populations, and, where appropriate, to consider multidisciplinary approaches to care.

### Highlights

- TMDs are prevalent in FMS patients, affecting 3 out of 4 individuals.
- Most FMS patients present with a myogenous TMD condition.
- Patients seeking treatment for painful TMDs appear more likely to have CWP or FMS.

## Figures and Tables

**Appendix Fig 1:** Meta-analysis of prevalence of FMS in patients with TMDs (descriptive cross-sectional studies). Note: Weights reflect those derived from application of random-effects meta-analysis.

**Table:** 

Study	Sample size	FMS (n)	Weight (%)	Percentage (95% CI)	Proportion(95% CI)
Aaron et al 2000	25	3	24.35	12.00 (2.55 to 31.22)	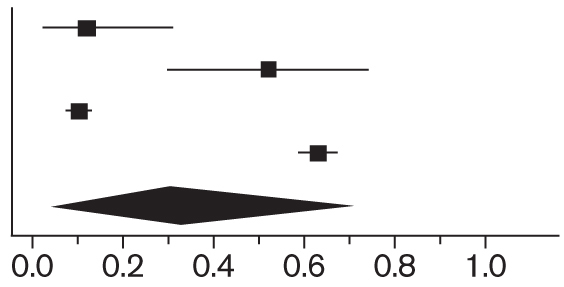
Leblebici et al 2007	21	11	24.10	52.38 (29.78 to 74.29)	
Velly et al 2010	572	60	25.78	10.49 (8.10 to 13.30)	
Losert-Bruggner et al 2018	555	351	25.77	63.24 (59.08 to 67.27)	
Total	1173	425	100	32.65 (4.52 to 71.02)	

Heterogeneity: Q(3) = 399.94; p < 0.001; I^2^ = 99.3% (95% CI = 98.9 to 99.5)

**Appendix Fig 2:** Meta-analysis of prevalence of TMDs in patients with FMS. Note: Weights reflect those derived from application of fixed-effects meta-analysis.

**Table:** 

Study	Sample size	TMD (n)	Weight (%)	Percentage (95% CI)	Proportion(95% CI)
Rhodus et al 2003	67	45	18.00	67.16 (54.61 to 78.15)	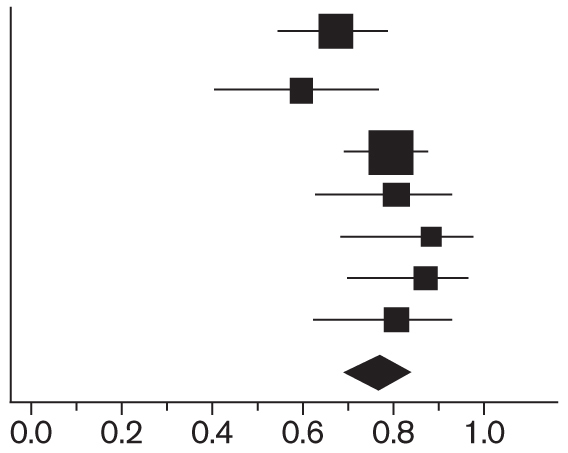
Balasubramaniam et al 2007	32	19	12.84	59.38 (40.65 to 76.30)	
Salvetti et al 2007	93	74	20.11	79.57 (69.95 to 87.23)	
Leblebici et al 2007	31	25	12.63	80.65 (62.53 to 92.55)	
Silva et al 2012	25	22	11.17	88.00 (68.78 to 97.45)	
Gui et al 2013	31	27	12.63	87.10 (70.17 to 96.37)	
Venere et al 2015	31	25	12.63	80.65 (62.53 to 92.55)	
Total	322	237	100	76.77 (69.53 to 83.31)	

Heterogeneity: Q(3) = 399.94; p < 0.001; I^2^ = 99.3% (95% CI = 98.9 to 99.5)

**Appendix Fig 3:** Meta-analyses of prevalence of (a) muscle, (b) disc displacement, and (c) inflammatory-degenerative TMDs in patients with FMS (descriptive cross-sectional studies). Note: Weights reflect those derived from application of fixed-effects (inflammatory-degenerative) and random-effects (muscle, disc displacement) meta-analyses.

**Table:** 

Study	Sample size	FMS (n)	Weight (%)	Percentage (95% CI)	Proportion(95% CI)
Balasubramaniam et al 2007	32	14	15.96	43.75 (26.36 to 62.34)	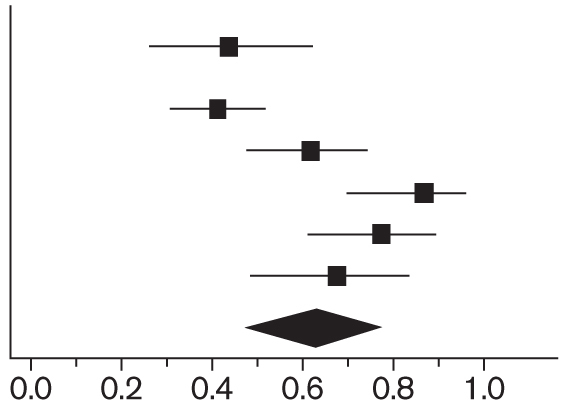
Salvetti et al 2007	93	38	18.22	40.86 (30.77 to 51.55)	
Fraga et al 2012	60	37	17.50	61.67 (48.21 to 73.93)	
Gui et al 2013	31	27	15.87	87.10 (70.17 to 96.37)	
Pimentel et al 2013	40	31	16.58	77.50 (61.55 to 89.16)	
Venere et al 2015	31	21	15.87	67.74 (48.63 to 83.32)	
Total	287	168	100.00	63.10 (47.67 to 77.27)	

Heterogeneity: Q(5) = 34.96; p < 0.001; I^2^ = 85.7% (95% CI = 70.9 to 93.0)

**Table:** 

Study	Sample size	FMS (n)	Weight (%)	Percentage (95% CI)	Proportion(95% CI)
Balasubramaniam et al 2007	32	4	11.26	12.50 (3.51 to 29.00)	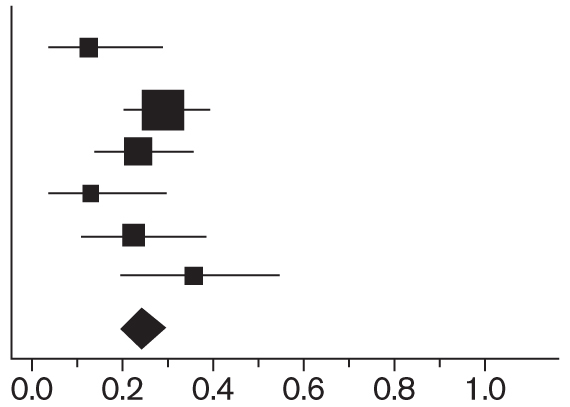
Salvetti et al 2007	93	27	32.08	29.03 (20.08 to 39.36)	
Fraga et al 2012	60	14	20.82	23.33 (13.38 to 36.04)	
Gui et al 2013	31	4	10.92	12.90 (3.63 to 29.83)	
Pimentel et al 2013	40	9	13.99	22.50 (10.84 to 38.45)	
Venere et al 2015	31	11	10.92	35.48 (19.23 to 54.63)	
Total	287	69	100.00	24.20 (19.41 to 29.52)	

Heterogeneity: Q(5) = 8.01; p = 0.156; I^2^ = 37.6% (95% CI = 0.0 to 75.2)

**Table:** 

Study	Sample size	FMS (n)	Weight (%)	Percentage (95% CI)	Proportion(95% CI)
Balasubramaniam et al 2007	32	15	16.30	46.88 (29.09 to 65.26)	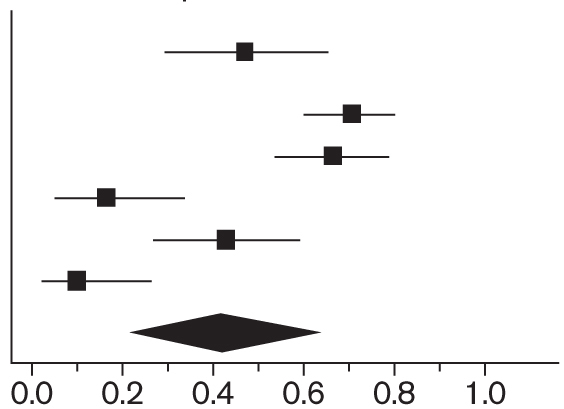
Salvetti et al 2007	93	66	17.46	70.97 (60.64 to 79.92)	
Fraga et al 2012	60	40	17.10	66.67 (53.31 to 78.31)	
Gui et al 2013	31	5	16.25	16.13 (5.45 to 33.73)	
Pimentel et al 2013	40	17	16.63	42.50 (27.04 to 59.11)	
Venere et al 2015	31	3	16.25	9.68 (2.04 to 25.75)	
Total	287	146	100	41.79 (21.93 to 63.15)	

Heterogeneity: Q(3) = 399.94; p < 0.001; I^2^ = 99.3% (95% CI = 98.9 to 99.5)

## Appendices

**Appendix Table 1:** Guideline for the Newcastle-Ottawa Quality Assessment Scale To Assess Quality and Bias of Studies

	Star awarded criteria		
	Case-control study	Cross-sectional study	Cohort study
Selection	Independent validation for cases (*)	Representative of the average in the target population and sampling method described (*)	Representative of the exposed cohort in the community (*)
	Obviously representative series of cases (*)	Sample size ≥ 100 (*)	Non-exposed cohort selected from the same community as the exposed cohort (*)
	Community controls (*)	Non respondents described and same rate for both groups (*)	Assessment blinded to case/control status and/or secure record (*)
	No history of disease for controls (*)	Validated measurement tool (**), Non-validated but described (*)	No outcome of interest at start of study (*)
Compatibility	Study controls for most important factor (*), for any additional factor (*)	Study controls for most important factor (*), for any additional factor (*)	Study controls for most important factor (*), for any additional factor (*)
Outcome	Assessment blinded to case/control status and/or secure record (*)	a) Independent blind assessment (**) b) Record linkage (**) c) Self-report (*)	a) Independent blind assessment (**) b) Record linkage (**) c) Self -report (*)
	Same method of ascertainment for cases and controls (*)	Clearly described and appropriate, association is presented, including confidence intervals and the probability level (*P* value) (*)	Follow-up long enough for outcome evaluation (*)
	Non respondents described and same rate for both groups (*)		Complete follow-up for all subjects (*)

Scores are given out of a total of nine stars.
